# The Provision of Spiritual Care in Hospices: A Study in Four Hospices in North Rhine-Westphalia

**DOI:** 10.1007/s10943-017-0396-y

**Published:** 2017-04-25

**Authors:** Andreas Walker, Christof Breitsameter

**Affiliations:** Katholisch-Theologische Fakultät, Lehrstuhl für Moraltheologie, Ludwig-Maximilians-University Munich, Geschwister-Scholl-Platz 1, 80539 Munich, Germany

**Keywords:** Nursing ethics, Hospice care, End-of-life decision-making, Palliative care, Spiritual care

## Abstract

This article considers the role and the practices of spiritual care in hospices. While spiritual care was firmly established as one of the four pillars of practical hospice care alongside medical, psychological and social care by Cicely Saunders, the importance and functions of spiritual care in daily practice remain arguable. When speaking about spirituality, what are we actually speaking about? What form do the spiritual relations take between full-time staff and volunteers on the one hand, and the patients and their family members on the other? These were central questions of a qualitative study that we carried out in four hospices in North Rhine-Westphalia, Germany, to explore how spiritual care is provided in hospices and what significance spirituality has in hospices. The study shows that the advantages of a broader definition of spirituality lie in “spiritual care” no longer being bound to one single profession, namely that of the chaplain. It also opens the way for nurses and volunteers—irrespective of their own religious beliefs—to provide spiritual end-of-life care to patients in hospices. If the hospice nurses and volunteers were able to mitigate the patients’ fear not only by using medications but also in a psychosocial or spiritual respect, then they saw this as a successful psychological and spiritual guidance. The spiritual guidance is to some degree independent of religious belief because it refers to a “spirit” or “inner core” of human beings. But this guidance needs assistance from professional knowledge considering religious rituals if the patients are deeply rooted in a (non-Christian) religion. Here, the lack of knowledge could be eliminated by further education as an essential but not sufficient condition.

## Introduction

Cicely Saunders, the founder of the modern hospice movement, ensured that spirituality was firmly established as one of the four pillars of practical hospice care alongside medical, psychological and social care. In developing her concept of spirituality, Saunders was influenced by Viktor Frankl (Walter 1996). Frankl developed logotherapy, a form of therapy based on the belief that patients should be able to give meaning and purpose to their lives. If this meaning is absent, people suffer from existential distress (Frankl 1985). In her article entitled “Spiritual Pain”, Saunders utilizes Frankl’s concept of meaning to approach the question of spirituality: “[W]e can always persevere with the practical. Care for the physical needs; the time taken to elucidate a symptom, the quiet acceptance of family’s angry demands, the way nursing care is given, can carry at all and can reach the most hidden places. Though this may be all we can offer to inarticulate spiritual pain, it may be enough as our patients finally face the truth on the other side of death” (Saunders 2006, p. 221). In her concept of “total pain”, Cicely Saunders was well aware of the need to understand spirituality in more than just the religious sense, even though she herself was from a Christian background (Saunders 2006, pp. 71–77).

Murray and Zentner offer one of the most established definitions of spirituality. According to them, spirituality is: “A quality that goes beyond religious affiliation, that strives for inspiration, reverence, awe, meaning and purpose, even in those who do not believe in any god”. The spiritual dimension increasingly comes into focus, “when the person faces emotional stress, physical illness or death” (Murray and Zentner 1989, p. 259). Spirituality, Tanyi notes, improves the individual quality of life by yielding hope and joy and by helping to accept one’s own mortality (Tanyi 2002). At a conference in the year 2009, where “over forty US leaders in palliative care, as well as spirituality and theology” were present, spirituality was defined as “the aspect of humanity that refers to the way individuals seek and express meaning and purpose and the way they experience their connectedness to the moment, to self, to others, to nature, and to the significant or sacred” (Puchalski 2010). This definition conceives spirituality as a broader concept than religion. Spirituality can have to do with beauty, values, altruism, idealism and “awareness of the tragic” (Cohen et al. 2012, pp. 801–802).

The unignorable impact of spiritual well-being on the quality of patients’ lives is frequently being discussed and has been illustrated by various studies. Hamilton emphasized the positive impact of the spiritual dimension on healing and health (Hamilton 1998). Sherman et al. have shown that the quality of life of patients with advanced cancer and AIDS can be increased if healthcare practitioners recognize and tend to the spiritual needs of patients and their relatives. They can supportively stand by their side with prayers, intimacy, and active listening in the face of losses that come with terminal illness and imminent death (Sherman et al. 2005). Giordano and Engebretson have stressed that spiritual experiences are a neurocognitive phenomenon with a salutogenic effect and should therefore be facilitated by healthcare professionals (Giordano and Engebretson 2006). In a recent study, Baldacchino et al. (2012) accentuated the importance of patients’ spiritual coping with life-threatening illnesses. The reflection on spiritual pain shows an original connection to physical suffering in a way that less spiritual pain can mean to improve physical well-being.

However, it remains disputable if and how spiritual needs of patients in hospices can be tended to and if religious and spiritual notions coincide. Dudley et al. (1995) point out that spirituality encompasses needs und forms of belief independent from organized religion. This aspect stresses the importance of interdisciplinary teams in hospices to meet the spiritual, but not necessarily religious needs of patients with meditation, music therapy and conversations. Prior to Dudley et al., O’Connor noted that spiritual needs may range from religious rituals to secular experiences, as they can occur with music or poetry. The challenge for healthcare professionals then consists in supporting patients by offering “an environment that recognizes individual needs and attempts to reduce fears” (O’Connor 1988, p. 37).

## Description of the Problem and Purpose

Since 2010, we have been conducting a qualitative research about conflicts and decision-making in hospices in North Rhine-Westphalia, Germany. This research encompassed issues like moral conflicts between nurses, questions of responsibility or reflections on ethical decisions. Since 2012, our research focused on questions relating to spirituality as well. We wanted to know what type of role spirituality plays in daily practice, how spiritual care is provided considering the holistic approach of hospice care, and how spirituality is understood by full-time staff and the volunteers. Even if spirituality is perceived as a broader concept than religion, as mentioned in the introduction, it is necessary to consider religious questions in our research because hospices in Germany are full of religious symbols. These symbols are mostly Christian, Islamic or Buddhist, and they are an integral part of the understanding of the spirituality of the hospices. Beyond the religious topic, we wanted to know how spirituality is supported. Therefore we have structured our research in four items: (1) How do the hospice’s full-time staff and volunteers understand spirituality? (2) What rituals and symbols do hospices provide to support spirituality and/or religious practices? (3) Is religious diversity an issue? (4) What does spiritual care look like in practice?

## Methods

The research questions required a qualitative design. Data collection was predominantly based on interviews. Data collection and evaluation of our study since 2010 has followed the methods of grounded theory, which was developed by Barney G. Glaser and Anselm L. Strauss and modified by Juliet Corbin and Strauss, because it abstains from presupposed theoretical concepts, and therefore does not merely verify hypotheses, but generates them (Glaser and Strauss 1967; Corbin and Strauss 2008). The logical structure of this procedure can be described, in the words of the American philosopher Charles Sanders Peirce, as abduction: “Abduction is the only logical operation which introduces any new idea; for induction does nothing but determine a value, and deduction merely evolves the necessary consequences of a pure hypothesis” (Peirce 1934, p. 362). Abduction allows for the factor of surprise in the acquisition of data.

### Participants

Between April and May 2012, 22 semi-structured interviews were conducted with full-time hospice staff, including nurses (5), the directors of patient care (2), members of the psychiatric service (3), the directors of the hospices (1), chaplains (2) and volunteers (9) of four hospices in North Rhine-Westphalia (Table 1). The selection of participants was made according to two criteria: (a) willingness to take part in our study, and (b) experience with spiritual end-of-life care. In German hospices, volunteers are often more responsible for the social and therefore psychological comfort of the patient than the nursing staff. This explains the relatively large number of volunteers in our study. On the other hand chaplains are the professionals in regard to religious questions, e.g. the afterlife, but they are generally not hospice employees. Hence, we have just two chaplains in our study, because one hospice was managed by a chaplain (Protestant) and he consulted a second one (Roman Catholic) from a nearby hospital. Because nurses and volunteers are primarily women, care was taken to include male nurses and volunteers in our interviews to generate a diversified image of our object of research.

**Table 1:** Participants

Interviewees	Nurses	Directors of hospice	Psychiatric service	Chaplains	Director of patients care	Volunteers
16 female/6 male	4 female/1 male	1 (female)	3 female	2 male	3 female	6 female/3male

### Question Guidelines

The initial question of the interviews was directed at the interviewee’s training and motivation for working in a hospice and his or her spiritual/religious belief. Most of the interviewees (19) had a religious (Roman Catholic or Protestant) background (8 female/3 male of the full-time staff, 5 female/3 male of the volunteers). Some of them (2) called themselves religious but did not want to get involved with a church. Others called themselves “spiritual” (2 female of the full-time staff, one female volunteer). Eight members of the full-time staff (5 female, 3 male) had a further education concerning spiritual questions or questions of mourning (Table 2). Three female volunteers had a spiritual education. This background information was necessary to provide context for the interviewee’s attitude towards spiritual end-of-life care. The next question referenced the concept of spirituality. Rooms, rituals and symbols of religion and spirituality were a topic as well as the religious diversity in a hospice. Subsequently, the interviewer raised the issue of offering spiritual care to patients and their relatives and the needs of the spiritual/religious needs of patients. The questions were arranged according to the following pattern: subjective belief—range of spiritual options in the specific institution—daily practice. Member checks were applied as interviewers occasionally referred to core statements of the interviewees for clarification and to ensure their validity.

**Table 2:** Religious affiliation of the participants

	Religious background	Spiritual	Spiritual educated
Full-time staff (13)	8 female/3 male	2 female	5 female/3 male
Volunteers (9)	5 female/3 male	1 female	3 female

### Ethical Considerations

The Ethics Commission (Ethics Commission of the Medical Faculty of the Ruhr-University, Bochum, 21.10.2010, Registration-No.: 3850-10) raised no objections following the submission of the study protocol. The research was approved by the hospice directors, the chaplains, the nursing teams and the volunteers. One of the scientists explained the study verbally to the participants, and they received an information letter about the study as well. After this, they were asked to sign a consent form. Informed consent was obtained from all individual participants included in the study.

### Data Collection and Analysis

The interviews were conducted by one of the authors (Walker) and two social scientists (Valerie Grimm and Kristin Illiger). They were recorded using a digital recording device and then transcribed verbatim. During this process, the data were anonymized and are now being stored at the institution responsible for the research. For the process of transcription, we used the EXMARaLDA computer programme. The two authors working independently from one another conducted the encoding process.

The results were compared and discussed in regular meetings. Due to our different professional backgrounds, one of us is a theologian, the other a philosopher, different priorities were set occasionally in the course of evaluation. This rendered necessary further interviews to clarify remaining questions. The process of data collection was concluded after it had been established that no further categories were to be identified, and it could be said, in relation to the underlying research questions, that a state of theoretical saturation had been reached.

## Results

The following results are presented according to the key questions asked, namely (1) the concept of spirituality, (2) rooms, rituals and symbols in hospices, (3) religious beliefs and diversity practices in hospices, (4) supporting spirituality, and (5) successful spiritual end-of-life care (Table 3).

**Table 3:** Results concerning the provision of spiritual care in hospices

Issue	Results
The concept of spirituality	No homogenous concept of spirituality Spirituality includes religious beliefs as well as a free-spirit that exceeds religion Every patient understands spirituality as he or she likes
Rooms, rituals and symbols in hospices supporting spiritual end-of-life care	Silent room or chapel Special forms of therapy (aroma or music therapy) Christian symbols like crosses Sacraments Prayers and communal singing Personal belongings (like pictures that have a spiritual meaning)
Religious beliefs and diversity practices in hospices	Limited knowledge by full-time staff and volunteers regarding religious affiliations that were different than their own Especially when treating moslems they are afraid of doing something wrong
Practices supporting spirituality	Meditation Establishing trust and nearness Giving the opportunity to talk Listening Singing or praying together Offering alternative forms of therapy
Successful spiritual end-of-life care	To take away someone’s fear Acceptance of one’s own death independent of any particular religion or belief

### The Concept of Spirituality

In the interviews, the concept of spirituality proved to be highly indistinct. One person said: “spirituality […] is difficult to grasp”; another said it was “another area where everything that has a religious aspect is part of it”. It had something to do with “the spirit” and was the “spiritual side of a person”. Some staff members included God in their definitions, while for others it was something “free-spirited, rather than fixed” in its meaning. Understanding spirituality as something that on one hand includes God, and exceeds religion on the other, corresponds with a study by McSherry et al. At the same time, the authors warn that not even the Christian understanding of spirituality is necessarily homogenous (McSherry et al. 2004, p. 938). But, as one of the interviewees told us “[e]verybody is spiritual whether they are aware of the fact or not”.

One volunteer member of staff expressed the following opinion when commenting on the status of spirituality in the everyday practice of the hospice: “My impression is that spirituality, although it is clearly wanted in hospices […] and is in the statutes as one of the aims, I mean one of four or five aims we should be trying to fulfill, in practice it is not given the importance you might think. One reason for that is of course that we haven’t been able to agree on a definition”. However, it is questionable whether the importance of spirituality in the everyday practice of hospices is dependent on there being an agreed definition. One core objective of a hospice lies in trying to take the greatest possible account of a patient’s wishes, thus precluding any definition which might prove to be too narrow. Every patient is given the space to understand spirituality as he or she likes (Stirling 2007). Milligan also emphasizes this position when he writes: “Spirituality should always be regarded as unique to the individual” (Milligan 2011, p. 48). This approach is hardly conducive to finding a definition which seeks to include all possible aspects of the concept. According to the European Association for Palliative Care, these aspects include “existential challenges” as well as “value-based considerations and attitudes” and “religious considerations and foundations” (EAPC taskforce on Spiritual Care in Palliative Care 2010). This coincides with Bash’s position that “spiritual experience is what each person says it is” and that “the task of nurses is to identify and respect that person’s expression of their spiritual experience and to offer them appropriate support” (Bash 2003, p. 14).

### Rooms, Rituals and Symbols in Hospices

Hospices usually provide a so-called room of tranquility which the patients and their family members are able to use as a place of contemplation and prayer. Alternatively, hospices have a chapel into which a prayer mat and a Koran for use by Muslim patients have been placed. The patients’ rooms are often sparsely decorated, devoid of any crosses or other religious symbols; however, if the hospice specifically identifies itself as a Christian institution, there are crosses in the rooms that will be removed at the patient’s request. Occasionally, the pictures hanging in the entrance hall, in the lounge, and in the corridors also have “a kind of spiritual character because they have a kind of levity, a sort of resonance”. Even when Christian symbols are in use, the hospices emphasize their openness to people of all faiths.

The spatial layout of a hospice is intended to achieve “clarity and beauty”, creating a “very special atmosphere” by being peaceful and well-lit. This provides the basis for a range of services available to the patients, from discussions to prayers and communal singing to acts of religious worship and the sacraments (Holy Communion/the celebration of the Eucharist, Confession, Anointing of the Sick). Other types of therapy, such as aromatherapy and also music therapy, are used to supplement the spiritual side of care.

When a new patient is admitted to the hospice they are specifically asked about their religious affiliation, in order to enable the hospice to take better account of that patient’s (potential) spiritual needs. Patients often bring crosses, angels or pictures of the Virgin Mary with them as “sources of strength and comfort”; rosaries are requested especially by people entering the final stages of their lives. The patients’ choices of such items as crosses or pictures of the Virgin Mary clearly illustrates how the underlying spiritual conditions and the organization of rituals still follows the models laid down by Germany’s two largest churches, even though hospices have declared in principle that they are also open to non-Christians and atheists. This does not mean that spirituality does not take place in other contexts, as testified by the different specific types of therapy. However, the Christian context was present in the minds of the staff members we interviewed, acting as the background against which they would speak of spirituality in the sense of symbols and rituals.

### Religious Beliefs and Diversity Practices in Hospices

Spiritual diversity has been conceived as a challenge from early on. Hall points out that the patient’s interpretation of Catholicism doesn’t necessarily have to match that of the chaplain (Hall 1997). If hospices are using Christian symbols as a point of orientation, the question inevitably arises as to how they handle patients who are neither Roman Catholic nor Protestant, and whether they are able to cater to their spiritual needs.

When dealing with adherents of different religions, particular emphasis was laid on how important it was to be familiar with the specific religious practices so that the nurses would know how to react in any given situation. The nurses could access this kind of information by attending courses, by reading the relevant literature, or by learning from patients’ family members. In particular, in the case of Muslim patients, as our interviewees repeatedly stressed, family members would take over many of the tasks usually undertaken by the nursing staff, such as the cleansing of the dead body. Occasionally, a Muslim patient would request a hodja or imam to be called to his bedside. The chaplains openly admitted to having no contact with Muslim patients. One chaplain explicitly stated that he was “not responsible” for them. The nursing staff tended to be somewhat reticent when engaging with Muslim patients, because they were “mostly cared for by their families”. Consequently, they concentrated on the medical and nursing needs of the patients. When treating Muslim patients, a sense of “nervousness” sometimes arose in the team “because it’s such unknown territory for us that we’re scared of doing something wrong”.

In addition, the nursing staff said they were “hesitant” when dealing with people whose religious affiliations were different from their own, even if they had completed a relevant training course. One of our interviewees recounted her experiences with a Hindu patient, saying “Hinduism was completely new to me”. The chaplain had given her some information leaflets to read. Her main questions were: “What do we need to bear in mind? What is special about their set of beliefs?” It was precisely because of the nurses’ limited knowledge that it was important for them to show “respect for different religions” and to treat people with “sensitivity and humanity”.

Some of the volunteer staff also openly admitted the gaps in their knowledge: “I know that there are Muslims and that they have different rituals and a different way of seeing things, but I couldn`t tell you much about them”. Volunteer staff and chaplains alike tended to feel a greater sense of responsibility for atheists than they did for patients belonging to religions or denominations with which they were less familiar. We were told of one case in which the relationship between a chaplain and an atheist was established on the basis of music and poetry. “In some poems we talked to each other about, I openly expressed my feelings. For me that’s also akin to an encounter with God. […] The patient saw it differently. And both viewpoints existed side by side. And that was just fine”.

### Supporting Spirituality

One interviewee defined spirituality as an “attitude” governing the way in which people interact with each other. Another stated that spirituality lay in a “spirit of compassion”. It might be expressed through “little moments”, such the nurses gently laying their hands on the patients or passing them a glass of water. A further interviewee stated that spirituality was particularly helpful in “crisis situations”. “Patients’ finer spiritual needs and wishes develop here in the hospice when they realize that their lives are indeed slowly drawing to an end”. And it is against this backdrop that spirituality becomes connected to questions of a “life after death”.

Spiritual practices included “meditation”, people singing or praying together, or other alternative forms of therapy mentioned previously. But above all, patients were given the opportunity to talk. One nurse stated that she engaged spiritually with patients “when I treat the people, with whom I come into contact, with attentiveness and when I take their needs and wishes seriously, and when I show them respect […], when I can inspire a little trust in them”. The points which were identified by interviewees as being at the core of spiritual interaction between themselves and the patients were the ability to inspire trust and to be near at hand, but also to keep their distance when the situation required. “Spirituality for me doesn’t just mean praying and singing, […] but also being able to listen to and appreciate what a patient is saying to you about his own process of dying and about what comes after death. For me, those are the really spiritual conversations. There are certain spiritual practices, but in my work I am far less concerned with the practices and more with what takes place in the conversations, not only with the patients but also with the people who are closest to them […]”. Talking and listening therefore forms the basis of the spiritual relationship between patients and nurses, chaplains and volunteers. “Our primary task consists of appreciating and comprehending […] what he is feeling. Does he want to talk about death? Does he want to talk about dying? Is he in pain? I can gently try and find out these things by asking specific questions”. This does not mean, however, that every patient wishes to talk about dying and/or about topics of a spiritual nature. It is frequently the case that they just want “someone to be there” “in case things get serious”, someone who “will stay with them no matter what”. This description corresponds with the relational model of Callahan, which encompasses the communication of spiritual sensitivity through such means as “personhood, therapeutic touch, being present, listening” and “singing” (Callahan 2013, p. 175).

### Successful Spiritual End-of-Life Care

One interviewee described successful spiritual end-of-life care as follows: “The most important thing was always to take away someone’s fear, to take away their fear and to say: ‘I am with you.’” In our interviews, the hospice nurses and volunteer staff both stated that if they were able to mitigate the patients’ fear not only medicinally but also in a psychosocial or spiritual respect, they viewed this as a success. One person voiced the opinion that it was not particularly important whether someone was religious or not: it was more important that each patient is “happy” with his or her own attitude towards imminent death. In the final instance, a patient’s acceptance of their own death played a more important role than any particular system of belief, at least as far as the patient’s fear of death was concerned. The associations when dealing with spirituality pertain to the distress and fear of the dying person, their feelings of guilt and their memories, questions of “what is coming, the afterlife” and the manner in which the patient can bid farewell both to life itself and to the people they are leaving behind. “I cannot stop the patients dying and I cannot take away their distress, but I can be there for them and, through my presence, my compassion and my willingness to care for them I can take away a good deal of their fear”. This type of end-of-life care not only takes on a form of expressiveness through the ability to listen and talk, but also has silence as a core element—irrespective of whether spirituality is a topic of conversation, and whether it is shared or not.

Nevertheless, spirituality is only one aspect of everyday practice in a hospice. “There is nothing spiritual about someone suffering a sudden, chronic shortness of breath”. Attacks of this kind, together with other painful physical conditions, were clear indications that something specific needed to be undertaken to remove the patient’s source of pain. This raises the question of how much importance is ascribed to spirituality in the everyday practice of hospice care. “I think that, in practice, the aspect of spirituality doesn’t have the value placed on it that you might expect […] time is spent on more practical things like: ‘my arm hurts’ or ‘can you bring me a fresh cup of coffee?’ […] ‘Can you put me in a wheelchair and walk me around for a bit?’ A lot more time is given over to that kind of thing”. Of course, this may be connected to the fact that certain patients spend significantly shorter periods of time in hospices. If a dying person only spends three days or even a few hours in a hospice, then there is no time or space for any spiritual end-of-life care.

## Discussion

### Spirituality as a Vague Concept

The concept of “spiritual pain”, as Saunders developed it, remains largely indistinct. The open “definition” of spirituality has both advantages and disadvantages. Its breadth renders the concept virtually meaningless in itself, since everything that has some kind of meaning for human beings can be subsumed under it (Walter 1994; Paley 2008; Stephenson and Berry 2015). Nevertheless, the advantages of a broader—vague—definition lie in “spiritual care” no longer being bound to one single profession, namely that of the chaplain. It also opens the way for nurses and volunteer staff—irrespective of their own religious beliefs—to provide spiritual end-of-life care to patients in hospices (Walter 1997; Tanyi 2002; Stirling 2007). Of course, if spiritual care is bound to religious rituals like sacraments, chaplaincy professionals are requested (Carey 2012). But rituals like praying or singing can be shared with nurses or volunteers alike. This was confirmed within our study. In general, our findings are consistent with the “Nursing Diagnosis of Readiness for Enhanced Spiritual Well-Being” which is the “ability to experience and integrate meaning and purpose in life through connectedness with the self, others, art, music, literature, nature, or a power greater than oneself” (North American Nurses Diagnosis Association International 2009).

Referring to Hay, Narayanasamy attempts to show that spirituality is in fact grounded biologically, which is a materialistic foundation of a metaphysical claim. “Spirituality is rooted in an awareness which is part of the biological make up of human species. Spirituality is present in all individuals and it may manifest as inner peace and strength derived from perceived relationship with a transcendent God or an ultimate reality or whatever an individual values” (Narayanasamy 1999, p. 123). Moreover, Narayanasamy specifically associates terms such as love, hope and trust with spirituality, terms, which just as well express psychological relations. Spirituality “comes into focus particularly when an individual faces emotional stress, physical illness or death” (Narayanasamy 1999, p. 124). However, this does not necessarily have to be the case, as the interviewees pointed out. By no means does spirituality need to be present in all individuals, unless it merely expresses the ability to give meaning to something or to make aesthetic experiences. The metaphysical connotation of the attempts to define spirituality is unnecessary for grasping its practical relevance, as we have demonstrated in our study. To Swinton and Pattison, the value of an unassertive concept of spirituality lies in the fact that it can serve as a means of “sensitizing language” “to enhance human well-being”. This can happen in the search for hope, God or meaning (Swinton and Pattison 2010, p. 234).

### Spiritual Care Education

Wu and Lin point out that spiritual care education “has a positive impact on participants’ perceptions of spirituality and spiritual care” and suggest that a “higher education level and more spiritual care lessons or training courses can increase perception level” (Wu and Lin 2011, p. 255). Swinton and Pattison affirm that an education in the field of spirituality encompasses “a variety of human quests” and thus enables caregivers “to identify and respond effectively to the particular quests that encounter within whatever situation the nurse finds herself in” (Swinton and Pattison 2010, p. 235). Baldacchino entertains a similar view: “To enhance the development and practice of [the] nursing competencies, it is recommended that nurses should be provided with further continuing education on spiritual care to ameliorate their nursing care. These educational programs should also include sessions from hospital chaplains and members of the interdisciplinary team on self-awareness” (Baldacchino 2006, p. 894). In the same vein, Narayanasamy discusses the need for a special education for nurses concerning spiritual matters (Narayanasamy 2004). Wasner et al. suggest that spiritual training can generate “significant improvements in self-perceived compassion for the dying, but also in compassion for oneself” (Wasner et al. 2005, p. 103) and can help increase job satisfaction while reducing work-related stress.

By no means do we intend to deny that attentiveness, compassion and self-awareness are essential aspects of healthcare professions. But we cannot confirm that a special spiritual education is necessary for the hospice context by all means. Aren’t things like compassion and self-awareness preconditions for hospice work? Can they really be taught or trained? Or might this training in fact be merely a reminder of essential human properties? To quote a warning by Walter: If those “offering spiritual care to dying people” do not apply abilities exceeding the “listening skills to be found in any basic textbook on counselling […], spiritual care will be indistinguishable in practice from emotional/psychological care” (Walter 1997, p. 28). If “attentive listening”, “non-verbal communication” and “presence or giving time” are at the core of spiritual care, it can hardly be distinguished from psychological care, as Milligan points out (Milligan 2011, p. 53).

From the statements of interviewees we cannot conclude that caregivers or volunteers in hospices felt that they had been insufficiently prepared for the spiritual needs of patients. Most deemed spirituality a profoundly human property requiring sensitivity in human interactions. After all, patients are careful about with whom they share and talk about their faith, their experiences, worries and hardships. In general, the interviewees emphasized their openness towards the situations with patients as well as the fact that they were also permitted to define boundaries and discontinue conversations if patients pressure them with their own spiritual views. Spirituality is not something anyone could be compelled to and requires a certain convergence in communication. If relation-centred care is always already spiritual as Reblin et al. note (Reblin et al. 2014, p. 269), the caregivers and volunteers of our study have been well prepared. This applies to the institution of hospices we came upon. For a clinical environment, in which caregivers’ work is possibly less relation-centred, it may be useful to bring to mind the spiritual needs and to sensitize caregivers to this topic.

In general, most of the staff lacks knowledge concerning the rituals of different religions. It is certainly the case that the staff should be adequately qualified to cater appropriately to the spiritual needs of the patients, but our study has revealed that the staff does not and indeed cannot ensure an appropriate level of care due to the sheer diversity of religions represented in hospices. One solution is to call on trained experts to provide support, or to contact representatives of the religion in question—and in cases involving Muslim patients this is indeed standard practice, as confirmed in our interviews. Whether this also happens in cases involving smaller religions, we are unable to say. Professional training for the hospice staff, volunteers and chaplains regarding religion and spirituality could decrease this problem (McSherry et al. 2004; Baldacchino 2006; Van Leeuven et al. 2006).

## Conclusion

The hospice idea has at its core the principle of allowing people to die with dignity. This takes place in an atmosphere of peace and autonomy, with as little pain as possible and, to the greatest degree possible, in the circle of one’s most beloved family and friends. There are those who would suggest that none of this has anything to do with spirituality and that it is actually all about the psychosocial needs of the patients and residents, with it only being interpreted as spiritual by the staff. This is correct to a certain extent because the spiritual, in practice and as a consequence of its broad definition, does blend together with the psychosocial indeed, the former could not exist without the latter. But despite all the criticism surrounding the imprecise definition of spirituality, we have nevertheless attempted to show, that in the every day practice of hospices, this concept works as a form of tact to take away the fear of patients, regardless of how vague and multi-faceted it might be. Therefore, spiritual end-of-life care differs from clearly defined or at least more clearly definable areas such as physical or psychosocial care. Regarding spiritual conversations and spiritual practices, the content of the discussions and the practices are less important than the forms these practices take, which, in the final instance, are in the hands of the speaker or the person carrying out the spiritual practice. Due to their nature, they cannot be superseded or replaced by a physical or psychosocial discourse. Ultimately, people entering into spiritual discourse of this kind will find that there is a place for them within its open definition.

If hospice nurses and volunteer staff were able to mitigate the patients’ fear not only by administering medication to them, but also in a spiritual respect, they saw this as successful spiritual guidance. This is to some degree independent of religious belief because it refers to a “spirit” or “inner core” of human beings. But this guidance needs assistance from professional knowledge considering religious rituals if the patients are deeply rooted in a non-Christian religion. Here, the lack of knowledge could be eliminated by further education as an essential but not sufficient condition. This remains with the awareness and the attentiveness of the individual who performs spiritual end-of-life care, in regard to which it is not always possible to evaluate whether it is applied correctly or wrongly and whether it is successful. A rest of uncertainty remains.

## Limitations

Our study has been subject to a number of limitations. The individuals interviewed by us were aware of the importance of spirituality in day-to-day care. In particular, volunteers were in the pleasant position of being able to make sufficient time for patients and to experience spiritual moments with them. The results are therefore influenced by a specific setting and organizational structure. A different setting, in which time-management cannot be handled as generously, and in which volunteers do not play a significant role, is likely to generate different results. In hospices in Germany, the spiritual needs of patients are tended to as part of the caregiver’s daily practice. However, their perceptions are not entirely transferable to caregivers, for example, in hospitals. Data collection took place in small institutions and in only one German state. Results might therefore have been affected by our specific sample set inasmuch as the reactions of our interviewees to our questions or requirements might have been influenced by regional temperament or cultural background. In order to better understand spiritual end-of-life care, further studies are needed which involve hospital settings, palliative care units, nursing homes, and hospices with different religious backgrounds. Additionally, further studies are also required in institutions of different sizes and in different socio-cultural surroundings. These studies might be able to show us more specifically what spiritual care means and how it can be implemented in different institutions (Selman et al. 2014). Further research is also required concerning the question whether nurses and volunteers actually manage to meet the needs of patients and their family members in the care they provide. The lack of knowledge in these fields is apparent, as Cobb et al. recently pointed out (Cobb et al. 2012).
